# Ribosome Profiling and RNA Sequencing Reveal Genome-Wide Cellular Translation and Transcription Regulation Under Osmotic Stress in *Lactobacillus rhamnosus* ATCC 53103

**DOI:** 10.3389/fmicb.2021.781454

**Published:** 2021-11-25

**Authors:** Xuejing Fan, Tianyu Bao, Huaxi Yi, Zongcai Zhang, Kenan Zhang, Xin Liu, Xue Lin, Zhen Zhang, Zhen Feng

**Affiliations:** ^1^Key Laboratory of Dairy Science, Ministry of Education, College of Food Science, Northeast Agricultural University, Harbin, China; ^2^College of Food Science and Engineering, Ocean University of China, Qingdao, China; ^3^Spice and Beverage Research Institute, Chinese Academy of Tropical Agricultural Sciences, Wanning, China

**Keywords:** *L. rhamnosus*, ribosome profiling, osmotic stress, translation regulation, translation efficiency

## Abstract

To determine whether osmotic pressure affects the translation efficiency of *Lactobacillus rhamnosus*, the ribosome profiling assay was performed to analyze the changes in translation efficiency in *L*. *rhamnosus* ATCC 53103. Under osmotic stress, differentially expressed genes (DEGs) involved in fatty acid biosynthesis and metabolism, ribosome, and purine metabolism pathways were co-regulated with consistent expression direction at translation and transcription levels. DEGs involved in the biosynthesis of phenylalanine, tyrosine, and tryptophan, and the phosphotransferase system pathways also were co-regulated at translation and transcription levels, while they showed opposite expression direction at two levels. Moreover, DEGs involved in the two-component system, amino acid metabolism, and pyruvate metabolism pathways were only regulated at the transcription level. And DEGs involved in fructose and mannose metabolism were only regulated at the translation level. The translation efficiency of DEGs involved in the biosynthesis of amino acids was downregulated while in quorum sensing and PTS pathways was upregulated. In addition, the ribosome footprints accumulated in open reading frame regions resulted in impaired translation initiation and elongation under osmotic stress. In summary, *L. rhamnosus* ATCC 53103 could respond to osmotic stress by translation regulation and control the balance between survival and growth of cells by transcription and translation.

## Introduction

*Lactobacillus rhamnosus* Gorbach Goldin (LGG) as a probiotic strain and starter culture strain has been widely used in various types of fermented and functional food production. Furthermore, it also plays an important role in modern biotechnological fermentation processes (Moayyedi et al., 2018). The benefits of LGG were primarily in gastrointestinal health and immune function, including adaptation to the human intestinal environment and reproduction, prevention of colonization by pernicious organisms, regulation of human intestinal flora, prevention of diarrhea, relief of lactose intolerance, and improvement of intestinal function (Sun et al., 2019; Chandrasekharan et al., 2020; Song et al., 2020). Environmental stress is one of the greatest challenges to the production and enrichment of probiotic strains and starter culture strains, especially osmotic stress (Muruzovic et al., 2018). Osmotic stress appears with the accumulation of metabolites and continuous addition of sodium hydroxide neutralizing agent into the medium during high density culture of strains (Tian et al., 2014). Osmotic stress results in the water movement from inside the cell to the outside, changes in the cell volume intracellular solute concentration, dehydration of cells, and affects cell growth rate and metabolic activities (Lin et al., 2017; Tian et al., 2018). Osmotic stress influences the growth and robustness of probiotic strains and starter culture strains. Therefore, a clear understanding of how LGG responds to osmotic stress at the molecular level is crucial to improve the survival and growth of probiotic and starter culture strains in the fermentation process.

Many osmotic adaptation sophisticated mechanisms have been revealed at molecular and physiological levels in lactic acid bacteria (LAB). A series of anti-osmotic components, molecular chaperone proteins, genes acting as regulatory factors and sigma factors and transport proteins have been found to improve the osmotic adaptation of LAB. GroE-DnaK-DnaJ has a σ^A^ sigma factor promoter, which could bind the HrcA repressor to improve the adaptation of LAB under osmotic stress. F_0_F_1_-ATPase and K^+^-ATPase involve in response to the osmotic stress in LAB. Arginine, ornithine, arginine, and lysine also involve in the regulation of osmotic stress by the arginine deiminase pathway in LAB (Bucka-Kolendo and Sokolowska, 2017). Previous researches were focused on the expression regulations of genes at transcription level and the proteins (Palomino et al., 2016; Lv et al., 2017). The regulation of protein expression levels is vital for LAB in response to osmotic stress, since most cellular processes are catalyzed by proteins. Hence, regulation of gene expression at the translation level from the mRNA pool in response to osmotic stress are indeed valuable. At present, regulation of gene expression at the translation level is not fully understood in the adaptation to osmotic stress during the fermentation of LAB.

Translation from mRNA to protein depends on cellular factors and temporally coordinated transient interactions between tRNA and the ribosome, a two-subunit protein and RNA complex that orchestrates protein synthesis in the cell (Shcherbik and Pestov, 2019). Changes in gene expression levels of specific mRNA depend on the composition of the actively translating ribosome (Genuth and Barna, 2018). Translation efficiency (TE) is the metric of ribosome quality (Brandman et al., 2012). Stresses can trigger both translation initiation and elongation through different mechanisms (Wek, 2018). Ribosome profiling focuses on measuring ribosome occupancy, delineating translation regions precisely, revealing the genome’s full coding potential, and the regulation of genes expression at translation and transcription levels (Ingolia, 2016). It provides a quantitative, high-resolution translation profile, identifies previously unknown translation events through evaluating TE, and describes the specific features of translation-elongation (Lei et al., 2015). Using ribosome profiling, the reduction of 21 nt mRNA fragments (RPFs) and the formation or translocation of peptide were the major factors in the translation rate-limiting step that was revealed under hyperosmotic and oxidative stresses in *Saccharomyces cerevisiae* (Wu et al., 2019). The ribosome footprints accumulated in the initiation of open reading frame regions (ORFs) and the early translation elongation paused under heat stress in *Escherichia coli* (Zhang et al., 2017). However, there are barely studies on translation regulation under environmental stress in LAB using ribosome profiling, especially under osmotic stress.

The initial aim of this project was to reveal genome-wide cellular translation regulation under osmotic stress in *L. rhamnosus* ATCC 53103 by using RNA-seq and ribosome profiling, with the long-term goal of elucidating new strategies to increase the viability of LAB under environment stress.

## Materials and Methods

### Strain, Growth Conditions, and Osmotic Stress

*L. rhamnosus* ATCC 53103 was from the American Type Culture Collection (Manassas, VA, United States) and cultured in MRS medium at 37°C. It was revitalized in MRS medium at 37°C three times before use. Cultures were stored at –80°C in MRS medium containing 10% glycerol. The growth of *L. rhamnosus* ATCC 53103 was monitored by an automatic growth curve analyzer (Bioscreen Cpro; OY Growth Curves, Finland) at 600 nm (OD_600_). For osmotic stress, the cells (OD_600_ ∼1.8) were collected by centrifugation (6,000 × g, 10 min, 4°C) and then resuspended in MRS medium containing 0.2, 0.4, 0.6, and 0.8 M sodium lactate. The effect of osmotic stress on cell growth was further monitored by an automatic growth curve analyzer (Bioscreen Cpro; OY Growth Curves, Finland). The relevant results were shown in Supplementary Figure 1. Because a higher sodium lactate concentration (0.8 M) in the MRS inhibited the growth of *L. rhamnosus* ATCC 53103. When the sodium lactate concentration was 0.6 M, the growth rate of *L. rhamnosus* ATCC 53103 decreased significantly, while the viable counts reached 8.66 log_10_CFU/mL after 24 h culture. Therefore, the final concentration of sodium lactate in this study was 0.6 M.

### Total RNA Extraction and Library Construction

The cultures of control group and osmotic stress group were harvested by centrifugation (6,000 × g 15 min, 4°C) after 3.5 and 6.5 h (OD_600_ ∼1.0), respectively. Total RNAs were extracted using a TRIzol-based method (Life Technologies, CA, United States). rRNAs were removed using the Ribo-Zero Magnetic Gold Kit (Epicenter Biotechnologies, Madison, WI, United States). RNA quality was checked using the Agilent 2200 TapeStation system (Agilent Technologies, Inc., Santa Clara, CA, United States). The library was constructed using a TruSeq RNA Sample Prep Kit v2 (Illumina, San Diego, CA, United States), and then sequenced by using the Illumina HiSeqi^TM^ 2500 platform with pair-end 150 base reads.

### Ribosome Profiling

For Ribo-Seq, chloramphenicol (200 μM) was added into the cultures when OD_600_ reached 1.0, and then cells were harvested by centrifugation (6,000 × g, 10 min, 4°C) after shaking for 2 min. Bacterial sludge was washed using resuspension buffer (20 mL) composed of NH_4_Cl (100 mM), MgCl_2_ (10 mM), chloramphenicol (1 mM), and Tris-HCl (pH 8.0, 20 mM). The cells were collected by centrifugation (4,000 × g, 5 min, 4°C), and then were mixed immediately with cell lysis buffer. The resuspended extracts were transferred from lysis buffer to new microtubes, pipetted several times then incubated on ice for 10 min. The cells were triturated ten times through a 26-G needle. The lysate was collected by centrifugation (20,000 × g, 10 min, 4°C. To prepare RPFs, RNase I (7.5 μL) and DNase I (5 μL) were added to lysate (300 μL) to incubate for 45 min with gentle mixing. Nuclease digestion was stopped by adding RNase inhibitor (10 μL). RPFs were isolated using the RNA Clean and Concentrator-25 Kit (R1017, Zymo Research, Orange County, CA, United States). The Ribo-seq libraries were constructed using NEBNext^®^ Multiple Small RNA Library Prep Set from Illumina^®^ (catalog no. E7300S, E7300L). 140–160 bp PCR products were enriched to generate cDNA libraries and then sequenced using Illumina HiSeq^TM^ 2500.

### Analysis of Differentially Expressed Genes

The gene expression level was normalized by the fragments per kilobase of transcript per million (FPKM) of mapped reads to control the influence of gene lengths and amount of sequencing data in calculation of gene expression. The edgeR package^1^ was used to identify DEGs across sample groups. Gene ontology (GO) annotation and Kyoto Encyclopedia for Genes and Genomes (KEGG) pathway of DEGs were analyzed. According to expressions levels in translation and transcription, DEGs were classified into five different groups: unchanged (DEGs were not regulated at both two levels), homodirection (DEGs were regulated at the two levels with consistent trends), opposite (DEGs were regulated at the two levels with opposite trends), translation (DEGs were only regulated at the translation level) and transcription (DEGs were only regulated at the transcription level).

### Analysis of Differentially Expressed Gene Translation Efficiency and Its Correlation With Differentially Expressed Genes Expression at the Transcription Level

The TEs of all DEGs were identified, calculated, and compared using RiboDiff (Zhong et al., 2017). According to DEGs expression with the changes of TE at the transcription level, DEGs were classified into five different groups as described above: Unchanged; Homodirection; Opposite; TE (DEGs only had change at the TE level); and Transcription.

### Data Analysis

Raw data were filtered following the previous study (Langmead and Salzberg, 2012). Trimmed reads were mapped to *L. rhamnosus* reference transcriptome^2^ allowing no mismatches using Bowtie2 (version 2.2.8). Retained reads were aligned to the reference genome using Bowtie2. Genes and gene expression were identified and calculated using RSEM. Genes with|log_2_(fold change)| > 1 and a false discovery rate (FDR) < 0.05 were considered as significant DEGs. Both Ribo-seq and RNA-seq data types had two biological duplications.

## Results

### Analysis of Ribo-Seq and RNA-Seq Data

To systematically investigate the effect of osmotic stress on transcription and translation regulation, RNA-seq and ribosome profiling were analyzed in the same two sets of parallel populations of *L. rhamnosus* ATCC 53103 cells with two different concentrations of sodium lactate (control vs. 0.6 M). Each experiment was divided into control group (CG) and osmotic stress group (OS), respectively (Supplementary Figure 2). The regulation of transcription and translation under osmotic stress were examined by deep sequencing of cellular total mRNAs and RPFs, respectively. A large number of reads were produced by ribosome footprints and RNA-seq transcripts. The average read was around 30 bp.

The total reads, ranging from 11,356,406 to 81,788,555 per DNA library, were produced by deep sequencing (Supplementary Table 1). After filtering, around 30 and 20 million reads were generated from RPF of CG and OS samples, respectively. Around 26 and 24 million mRNA-seq reads were generated from CG and OS samples, respectively. These reads were mapped to *L. rhamnosus* reference transcriptome data. The mapping efficiency of RPF samples was ∼20%. There are high correlations between the two biological duplication (*R*^2^>0.85) for both Ribo-seq and RNA-seq data. A total of 5578 and 5616 mapped genes were obtained from RPF samples of CG and OS, respectively. A total of 5,318 and 5,370 mapped genes were obtained from CG and OS mRNA samples, respectively.

**FIGURE 1:**
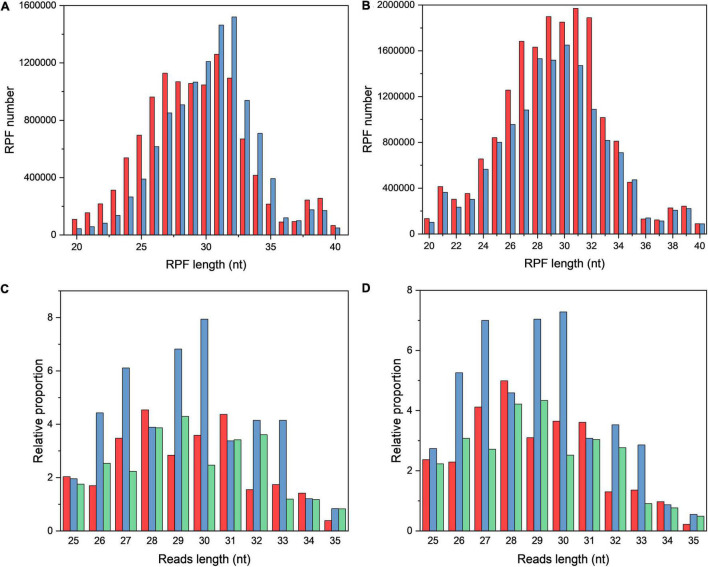
The characteristics of ribosome profiling data in OS and CG. **(A,B)** Length distribution of RPFs in OS **(A)** and CG **(B)**. The red and blue bars refer to two biological replicates. **(C,D)** Three-nucleotide periodicity at the first 35 nt of CDS in OS **(C)** and CG **(D)**.

The length of RPFs in OS and CG samples was around 30 nt (Figures 1A,B). The triplet periodicity between OS and CG samples were compared by scanning from the start codon to stop codon, and a strong three-nucleotide periodicity in OS and CG samples was observed (Figures 1C,D).

### Effect of Osmotic Stress on Expression and the Function of Differentially Expressed Genes at Translation and Transcription Levels

**FIGURE 2:**
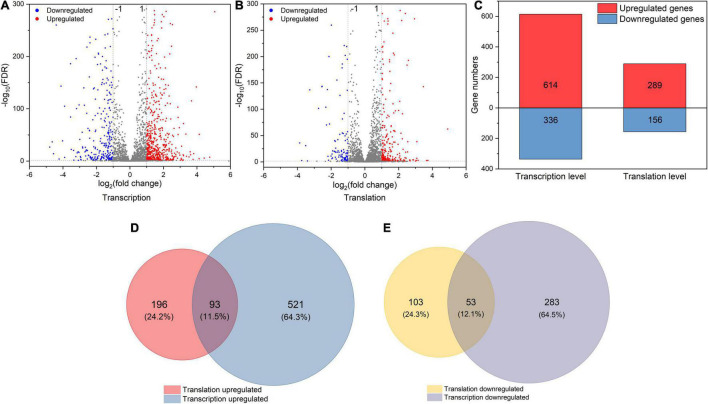
**(A,B)** The volcano plot of DEGs (|log_2_FC| > 1 and FDR < 0.05) at transcription and translation levels under osmotic stress. **(C)** The number of DEGs at transcription and translation levels under osmotic stress. **(D,E)** The relationship between osmotic responsive genes at transcription and translation levels. Genes analyzed in Figure 1 **(D,E)** were derived from Figure 1 **(A,B)**.

**FIGURE 3:**
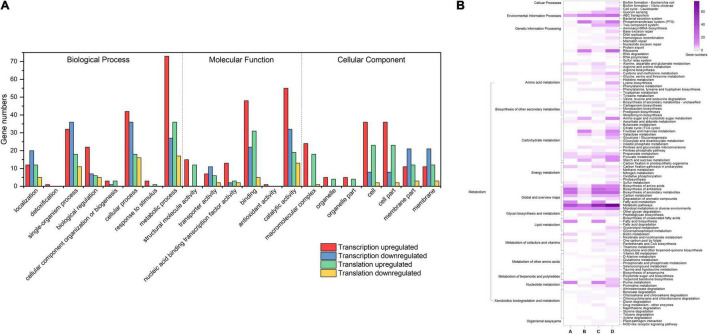
**(A)** GO analysis of DEGs in OS and CG. **(B)** KEGG pathways of DEGs in OS and CG.

To explore how *L. rhamnosus* ATCC 53103 cells deal with osmotic stress, both translation and transcription relative variations between CG and OS were examined. The results showed that osmotic stress changed gene expression at the translation and transcription levels (Figure 2). A total of 289 and 614 DEGs were upregulated while 156 and 336 DEGs were downregulated at the translation and transcription levels, respectively (Figures 2A–C). About a quarter (11.5 and 12.1%) of the regulated DEGs were shared between the two levels (Figures 2D,E). At the translation and transcription levels, the expression of genes from samples of OS was moderately correlated (*R*^2^ = 0.7757; Supplementary Figure 3). GO annotation and KEGG pathway analysis also showed that DEGs largely overlapped in GO and many KEGG pathways which also illustrated a big overlap at these two levels including ABC transporters, biosynthesis of antibiotics, secondary metabolites, and metabolic pathways etc. (Figures 3A,B). It is worthwhile to note that large numbers of DEGs showed different expression trends and many DEGs of enriched pathways showed a different response at the two levels. These results demonstrated that the regulation of DEGs at the translation level played an important role under osmotic stress in LGG.

### Effect of Osmotic Stress on the Dynamic Profiles of Translation and Transcription

**FIGURE 4:**
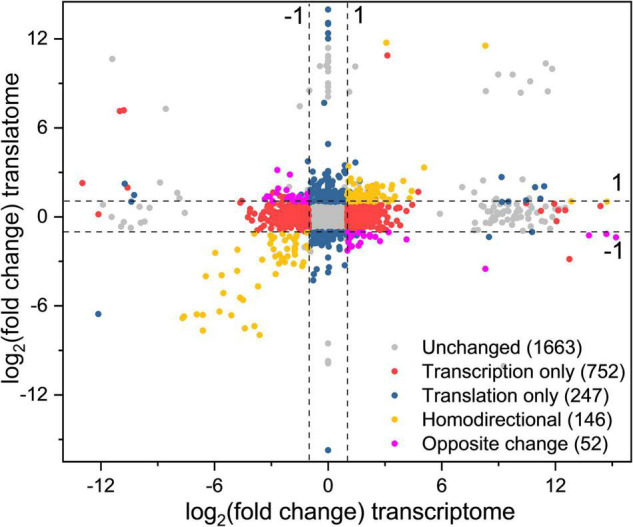
Simultaneous monitoring of the changes at the transcription and translation levels in *L. rhamnosus* ATCC 53103 under osmotic stress. Plot of the log_2_fold change of RPFs (OS/CG) against the log_2_fold change of respective mRNAs (OS/CG). The four dash lines indicate log_2_fold change values -1 or + 1.

To obtain the changes of gene expression trends at the translation and transcription levels simultaneously, DEGs were divided into different groups based on the fold change of gene expression (|log_2_(fold change)| > 1) between CG and OS (Figure 4 and Supplementary Table 2).

A total of 93 DEGs were upregulated at the two levels and 53 DEGs were downregulated at the two levels (yellow dots). These DEGs were mainly involved in the fatty acid biosynthesis and metabolism, ribosome assembly, and purine metabolism pathways. The DEGs *purD*, *purH*, *purN*, *purM*, *purQ*, *purC*, and *purK* involved in purine metabolism were downregulated from 99.37- to 249.25-fold and from 12.25- to 205.53-fold at the translation level and transcription level, respectively. The DEGs *accA*, *accD*, *accC1*, *accB*, *pksA*, and *fabH* involved in fatty acid biosynthesis were downregulated from 4.56- to 14.47-fold and from 3.52- to 7.35-fold at the translation level and transcription level, respectively.

A total of 24 DEGs were downregulated at the transcription level and upregulated at the translation level, while 28 DEGs were upregulated at the transcription level and downregulated at the translation level (purple dots). These DEGs were mainly involved in biosynthesis of phenylalanine, tyrosine, and tryptophan, protein export, phosphotransferase system (PTS), ABC transporters, galactose metabolism, and pyrimidine metabolism pathways. The DEGs *fruA* and *lacF* involved in the PTS pathway were upregulated and downregulated from 2.09- to 2.57-fold and from 7.63- to 8.43-fold at the translation level and the transcription level, respectively.

A total of 752 DEGs were regulated at the transcription level only (red dots). These DEGs were mainly involved in biosynthesis and metabolism of amino acid, the two-component system, ABC transporters and pyruvate metabolism pathways. The DEGs *tauA*, *tauB*, *tcyJ*, and *macB2* involved in the ABC transporters were upregulated from 4.00- to 13.64-fold, respectively. The DEGs *cydA*, *ciaR*, *dltC*, *citF*, and *citC* involved in the two-component system were downregulated from 2.26- to 8.07-fold, respectively.

A total of 247 DEGs were regulated at the translation level only (blue dots). The DEGs *levE*, *fruK*, *rhaD*, *sorA*, and *rhaB* involved in fructose and mannose metabolism were upregulated. The DEGs *licB*, *bglP*, *sorA*, *manX*, and *mtlF* involved in the PTS pathway also were upregulated. The DEGs *rplL*, *rpmD*, *rplF*, *rplN*, and *rplP* involved in ribosome assembly were upregulated about 2–3-fold. The DEGs *potA* and *potB* involved in the ABC transporters pathway were upregulated by 10.42- and 7.69-fold, respectively. Meanwhile, the DEGs *cysE*, *cysK*, *hisG*, *hisZ*, *nodI*, *oppA*, and *macB* involved in biosynthesis of amino acids and antibiotics, and ABC transporters pathway also were downregulated.

### Effect of Osmotic Stress on the Dynamic Profiles of Translation Efficiency and Transcription

**FIGURE 5:**
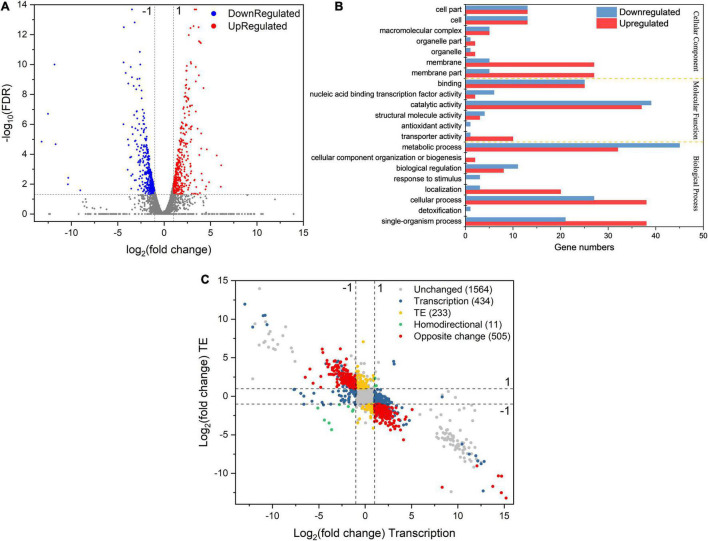
**(A)** The volcano plot of DEGs’ TE response under osmotic stress. **(B)** GO annotation of differentially expressed genes with largely changed translation efficiency under sodium lactate stress. **(C)** Simultaneous monitoring of the distribution of log_2_fold change TE and corresponding log_2_fold change mRNA abundance in *L. rhamnosus* ATCC 53103 under osmotic stress. The four dash lines indicate log_2_fold change values –1 or + 1.

TEs of 363 and 386 DEGs were upregulated and downregulated, respectively, which suggested that TE played an important role in response to osmotic stress in LGG (Figure 5A and Supplementary Table 3). GO annotation showed that these DEGs were mainly enriched in cell part, membrane part, binding, catalytic activity, metabolic process, biological regulation, localization, cellular, and single-organism process (Figure 5B). KEGG pathway analysis indicated that these DEGs were mainly involved in biosynthesis of secondary metabolites, biosynthesis of antibiotics, amino acids metabolism, pyruvate metabolism, carbon metabolism, purine metabolism, glycolysis pathway, ribosome assembly, the two-component system, ABC transporters, PTS pathways (Supplementary Table 4). The DEGs *rpsD*, *rpsT*, *rpmG*, *rpsQ*, *rpsC*, *rpsS*, *rpsJ*, *rpsZ*, *purD*, *purH*, *purN*, *purK*, *desR*, *yvfT*, *bceB*, *ywqE*, *iphP*, *htrA*, *opuCD*, *opuCC*, *opuCB*, and *opuCA* were involved in ribosome assembly, purine metabolism, the two-component system, and ABC transporters pathways, and their TEs were downregulated. Other DEGs, such as *secG*, *dppC*, *comA*, *yidC*, *manZ*, and *lacF* were involved in quorum sensing and PTS pathways and their TEs were upregulated.

**FIGURE 6:**
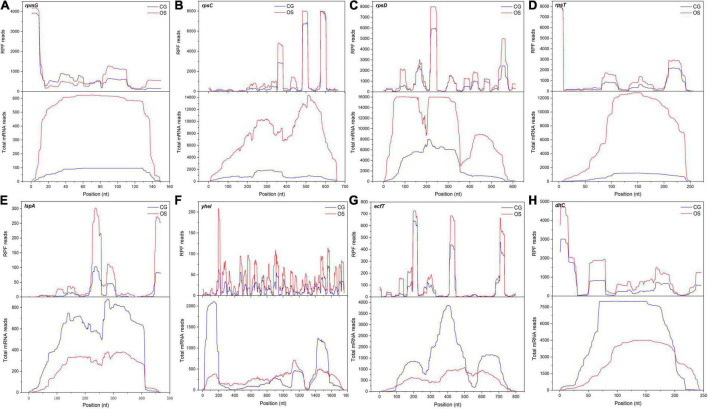
The mapped reads of DEGs with regulated TE and transcript under osmotic stress. **(A–H)** The DEGs were *rpmG, rpsC, rpsD, rpsT, lspA, yheI, ecfT* and *dltC*, respectively.

The calculation and analysis showed that the Pearson correlation coefficient between mRNA abundance and TE was –0.5845 (Supplementary Figure 4). This showed that transcription abundance affected TE. Hence, the distribution of DEGs was classified into different groups based on the regulation of TE and gene expression at the transcription level (Figure 5C). A total of 434 DEGs were regulated only at the transcription level while their TEs were not regulated, such as *rpsN*, *rplJ*, *accA*, *fabZ*, *pksA*, *tauA*, *tcyJ*, *nodI*, *purF*, and *trpA*. They were mainly involved in the assembly of ribosomes, fatty acid biosynthesis and metabolism, PTS, ABC transporters, and metabolism of starch, sucrose, purine, fructose, mannose, amino sugar, and nucleotide sugar pathways (Supplementary Table 5). TEs of 233 DEGs were regulated while their expressions were not regulated at the transcription level, such as *cysE*, *cysK*, *sdaAB*, *ilvE*, *tpiA*, *glcK*, *kdgK*, *rhaD*, and *ssdA*. They were mainly involved in the metabolism of cysteine and methionine, glycolysis, and microbial metabolism in diverse environment pathways (Supplementary Table 6). Only 11 DEGs were regulated at the transcription level, while their TEs were also regulated, and their regulations had similar trends. These DEGs included *fhs*, *purH*, *purN*, *accD*, *accB*, *purD*, *purK*, and *potC*. They were mainly involved in the metabolism of purine, fatty acid, propanoate, carbon, and pyruvate pathways (Supplementary Table 7). TEs of 286 DEGs were downregulated while their expressions were upregulated at the transcription level. These DEGs were mainly involved in pathways of biosynthesis and metabolism of amino acids, antibiotic biosynthesis, the citrate cycle, glycolysis, glutathione metabolism, carbon metabolism, amino sugar metabolism, nucleotide sugar metabolism, pyrimidine metabolism, and ribosome assembly. Such as the DEGs *dapB* and *dapH* were involved in lysine biosynthesis via the succinyl-DAP and acetyl-DAP pathways, respectively. *SpeF* was involved in arginine and proline metabolism. The DEGs *rpmG*, *rpsC*, *rpsD*, and *rpsT* were involved in ribosome functional group assembly. The mapped reads of these DEGs are shown in Figures 6A–D. TEs of 219 DEGs were upregulated and their expressions were downregulated at the transcription level. These DEGs mainly involved in pathways of protein export, ABC transporters, and two-component system (Supplementary Table 8). The DEGs included *lspA*, *yheI*, *ecfT*, and *dltC*, and the mapped reads are shown in Figures 6E–H.

### Effects of Osmotic Stress on Ribosomes Accumulation in Open Reading Frame Regions

**FIGURE 7:**
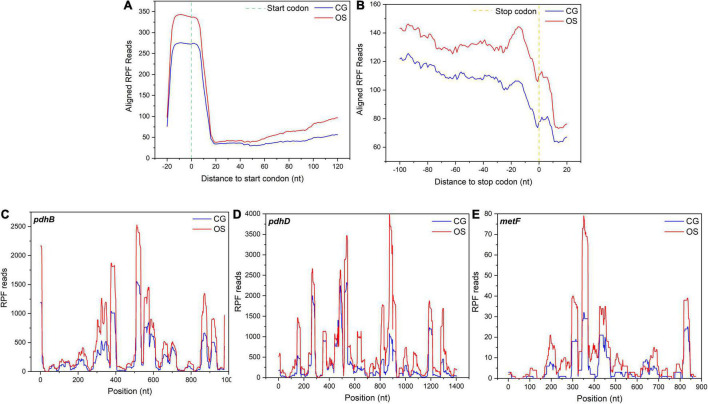
**(A)** Alignments of RPF reads in the 5′UTR of all mapped genes in a 20-nucleotide distance to start codon. **(B)** Alignments of the RPF reads in 3′UTR of all mapped genes in a 20-nucleotide distance to stop codon. **(C–E)** Three representative genes, *pdhB*, *pdhD*, and *metF*, with their ribosome footprint densities increased under osmotic stress in *L. rhamnosus* ATCC 53103.

Codon resolution is one of the advantages of ribosome profiling. To evaluate the translation events, the average occupancy of ribosomes was calculated around the start and stop codons. The footprints increased significantly under osmotic stress in *L. rhamnosus* ATCC 53103, which mapped to the initiation region around the 5′-end approximately at the –15 position (Figure 7A). In addition, ribosome occupancy from the nucleotide –100 to the end codon was also higher in OS than that in CG (Figure 7B). A few typical DEGs had higher ribosome occupancy under osmotic stress, such as *pdhB*, *pdhD*, and *metF* (Figures 7C–E). TEs of these DEGs were downregulated under osmotic stress.

## Discussion

The total reads were obtained from CG and OS samples, and the mapping efficiency of RPF samples was relatively low (∼20%) in this study. The present result was similar to *Saccharomyces cerevisiae* (16%) under starvation stress (Ingolia et al., 2009). It might be due to the large number of rRNAs that were removed. During translation, ribosome read density distribution moves three nucleotides simultaneously. Hence the representative feature of read density distribution is the triplet periodicity (Ingolia et al., 2009; Guo et al., 2010; Chew et al., 2013). The three-nucleotide periodicity were clearly observed in both CG and OS samples. The present result was similar with yeast, maize, and *Arabidopsis* under starvation, drought, dark, and sublethal hypoxia conditions, respectively (Ingolia et al., 2009; Chew et al., 2013; Liu et al., 2013; Juntawong et al., 2014; Lei et al., 2015).

Using RNA sequencing coupled with ribosome profiling, the changes of genome-wide gene expression were not only explored at the translation and transcription levels but also the interactions were revealed between both regulation levels under osmotic stress in *L. rhamnosus* ATCC 53103. Many DEGs were involved in fatty acid biosynthesis and metabolism, ribosomes, purine metabolism pathways, ABC transporters, and PTS pathways at the translation and transcription levels. As a stress sensor, ribosomes mediate the synthesis and attenuation of proteins in response to stress (Shcherbik and Pestov, 2019). The DEGs involved in ribosome assembly were upregulated under osmotic stress in *L. rhamnosus* ATCC 53103. It is essential for bacterial survival to strictly regulate fatty acid biosynthesis and metabolism (Yang et al., 2019). *FabH* catalyzes the type II fatty acid initiation biosynthesis reactions (Lai and Cronan, 2003). *FabZ* codifies a dehydratase, introduces double bonds into the carbon chain, and stimulates the production of unsaturated fatty acid (Siroli et al., 2020). However, these DEGs involved in fatty acid biosynthesis and metabolism pathways were downregulated at both levels. This reflected that *L. rhamnosus* needed to save cellular metabolic energy and increase environmental adaptation by limiting fatty acid biosynthesis (Adu et al., 2018). Purine metabolism was activated in the bacteria in response to environmental stress (Goncheva et al., 2019). As the genetic information transmitters and phosphate group donors, purine intermediates were not only involved in signal mediation but also ensured the living cell energy supply in response to environment stress. *L. rhamnosus* reduced growth rate under osmotic stress, and the DEGs involved in the purine metabolism pathway were downregulated at the translation and transcription levels. The synthesis of purine is energetically expensive (Peifer et al., 2012). The reduction of synthesis of purine maintained the stability of *L. rhamnosus* ATCC 53103 intracellular pool size, helped cells save energy, and keep cells growing slowly under osmotic stress (Shimaoka et al., 2007). Similarly, downregulated DEGs in the PTS pathway at the translation level also showed that conservation of energy was beneficial for *L. rhamnosus* ATCC 53103 in response to osmotic stress (Ganesan et al., 2007).

A total of 752 DEGs only were regulated only at the transcription level and 247 DEGs were regulated only at the translation level in the present study. These results indicated that *L. rhamnosus* ATCC 53103 regulated gene expression at the two levels independently under osmotic stress. Although translation regulation was not to generate new mRNA, it could be regarded as a rapid and direct environmental response (Sonenberg and Hinnebusch, 2009). Therefore, translation regulation plays a relatively independent and fine-tuning role in response to stress (Lackner et al., 2012). Genes regulated at the transcription level can be a barometer for the follow-up translation change (Lei et al., 2015). Genes upregulated only at the transcription level showed that *L. rhamnosus* ATCC 53103 regulated mRNA abundance to recover the TE reduction under osmotic stress. The ample mRNA provided a spare pool to facilitate the translation regulation immediately when osmotic stress abates in the future (Shenton et al., 2006). The interaction between the translation and transcription response boosts the gene expression flexibility, which makes for osmotic stress adaptation of *L. rhamnosus* ATCC 53103. Severe and rapid stresses might result in response quickly and independently of gene expression at translation and transcription levels; while moderate and chronic stresses might lead to more coordinated regulation at both levels (Lei et al., 2015).

The intracellular amino acid concentration is crucial for bacteria in response to osmotic stress (Gaucher et al., 2019). The DEGs involved in the amino acid biosynthesis pathway were upregulated at the transcription level under osmotic stress in the present study. A similar phenomenon was found under stress in other LAB (Papadimitriou et al., 2016). However, the reduction of TEs of the amino acid biosynthesis gene cluster resulted in the accumulation of uncharged tRNAs, lowering its activity and reducing the rates of overall protein synthesis (Spriggs et al., 2010). TEs of DEGs related to ribosome assembly were downregulated under osmotic stress, resulting in decreasing the freely available ribosome complexes for translation, affecting the mRNA translation, thereby reducing the production of protein and altering proteome allocation by the translation regulation (Song et al., 2018). This regulation would be crucial to the conservation of energy under osmotic stress. The TEs of DEGs involved in protein export, ABC transporters, and the two-component system were upregulated. The two-component system is made up primarily of a response regulator and histidine kinase, which associate with the cell membrane. LAB often regulates the expression of genes enriched in the two-component system to cope with environmental stress (Zhang et al., 2017). Osmotic stress showed a significant correlation with biosynthesis and metabolism of secondary metabolites and antibiotics. These changes might help *L. rhamnosus* ATCC 53103 to reprogram translation to adapt to the osmotic stress. The existence of various parallel mechanisms at the transcription and translation levels were to maintain the balance between survival and growth of *L. rhamnosus* ATCC 53103 under osmotic stress (Durfee et al., 2008).

Under osmotic stress, ribosome occupancy in the initiation region from the nucleotide –100 to the end codon increased dramatically. Furthermore, it was higher than that of the normal condition in the present study. It suggested that ribosomes pausing at initiation and later elongation steps was a general ribosome reaction under osmotic stress in prokaryotes (Shalgi et al., 2013). The accumulation of ribosomes resulted in increasing the ribosome density and the fraction of stalled bound ribosomes. This phenomenon would create traffic jams on all mRNAs and affect the TE (Zhang et al., 2017). Meanwhile, with the increase of bound ribosomes, the amount of tRNAs on mRNAs increased and the free tRNAs pool decreased. The reduction of free tRNAs pool would decrease translation initiation and elongation rates, reduce genes TE, and further retard the production of total protein (Shah et al., 2013). Therefore, translation dynamics of *L. rhamnosus* ATCC 53103 would change under osmotic stress. The global TE downregulation would provide another way to reduce the production of protein and promote survival of *L. rhamnosus* ATCC 53103 under osmotic stress.

## Conclusion

Based on ribosome profiling and RNA-seq, the study focused on a landscape of highly dynamic translation and transcription regulation, and revealed the changes of TEs of DEGs under osmotic stress in *L. rhamnosus* ATCC 53103. The DEGs involved in the purine metabolism pathway were downregulated at the translation and transcription levels under osmotic stress. The TEs of DEGs involved in fatty acid biosynthesis and metabolism pathways were not regulated, although these DEGs were downregulated at the translation and transcription levels. Abundant DEGs involved in the biosynthesis of amino acid pathways and ribosome assembly were upregulated at the transcription level and the TEs of these DEGs were downregulated. TEs of DEGs related to protein export, ABC transporters, and the two-component system were upregulated. The ribosome footprints accumulated in the ORF regions, resulting in impaired translation initiation and elongation under osmotic stress. *L. rhamnosus* ATCC 53103 controlled the balance between cell survival and growth by using transcription and translation parallel mechanisms under osmotic stress.

## Data Availability Statement

The data presented in the study are deposited in the Gene Expression Omnibus (GEO) repository, accession number GSE188929.

## Author Contributions

XF conceived and supervised the study. XF, TB, and HY designed the experiments. ZoZ, KZ, and XiL performed the experiments. XuL and ZhZ analyzed the data. XF and ZF wrote the manuscript. All authors have read and approved the manuscript.

## Conflict of Interest

The authors declare that the research was conducted in the absence of any commercial or financial relationships that could be construed as a potential conflict of interest.

## Publisher’s Note

All claims expressed in this article are solely those of the authors and do not necessarily represent those of their affiliated organizations, or those of the publisher, the editors and the reviewers. Any product that may be evaluated in this article, or claim that may be made by its manufacturer, is not guaranteed or endorsed by the publisher.

## Footnotes

1
http://www.rproject.org/

2
https://www.ncbi.nlm.nih.gov/nuccore/NC_013198.1

## Supplementary Material

Supplementary Figure 1 Effects of different sodium lactate concentration on the growth of *L. rhamnosus* ATCC 53103. **(A)** Growth curve of *L. rhamnosus* ATCC 53103 under different sodium lactate concentration. **(B)** The viable count of *L. rhamnosus* ATCC 53103 under different sodium lactate concentration.

Supplementary Figure 2 Overview of experimental design. RNA-seq and ribosome profiling were performed on CG and OS.

Supplementary Figure 3 Correlation of gene expression of mRNA abundance with Ribo-seq abundance of OS.

Supplementary Figure 4 Correlation of gene expression of mRNA abundance with gene translation efficiency of OS.
