# Supplementary figures and images for: Ribosome Profiling and RNA Sequencing Reveal Genome-Wide Cellular Translation and Transcription Regulation Under Osmotic Stress in Lactobacillus rhamnosus ATCC 53103
Source: Front Microbiol. 2021 Nov 25;12:781454. doi: 10.3389/fmicb.2021.781454 (PMC8656396; doi:10.3389/fmicb.2021.781454)

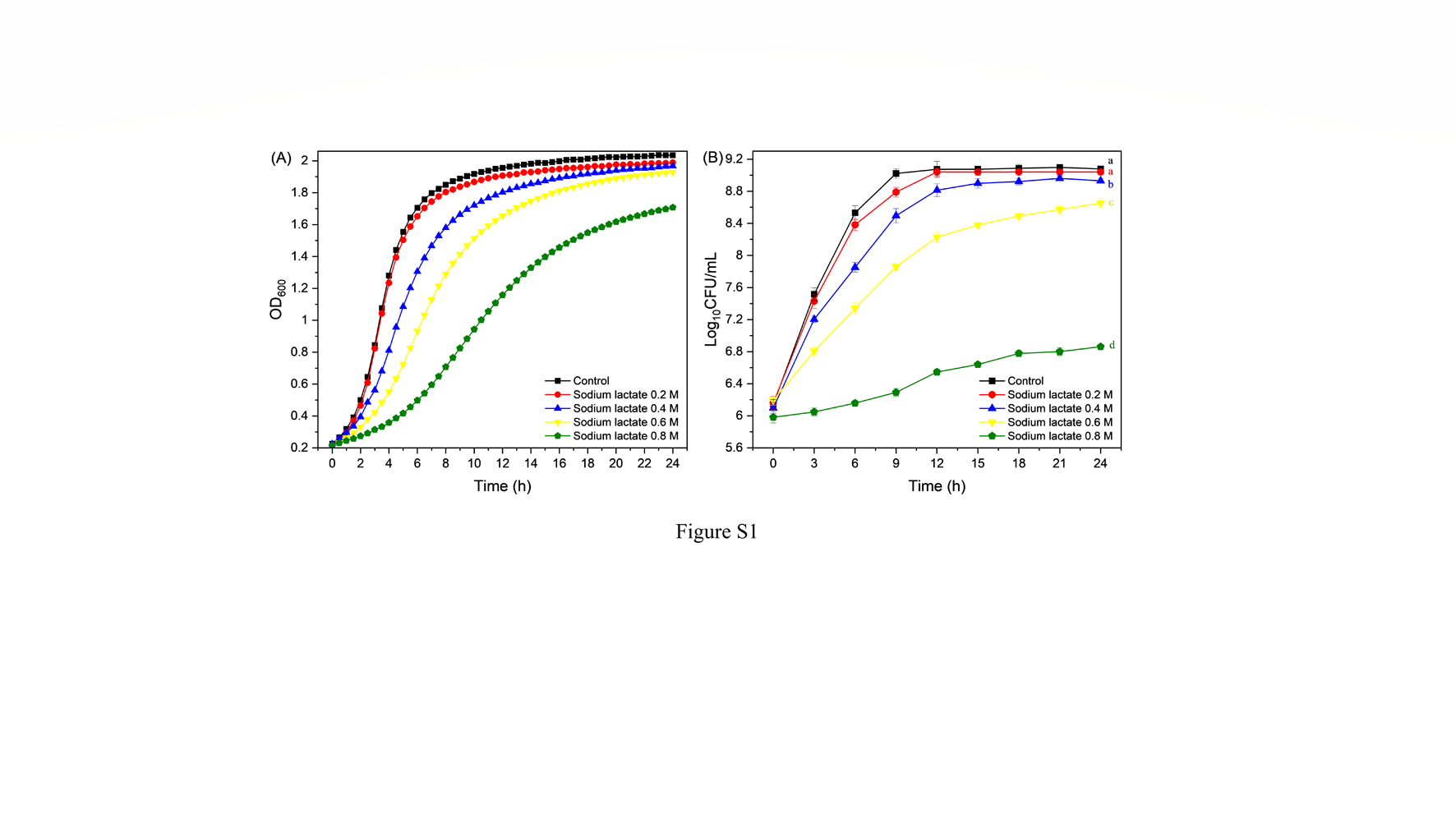

Supplement: Supplementary Figure 1 — Effects of different sodium lactate concentration on the growth of L. rhamnosus ATCC 53103. (A) Growth curve of L. rhamnosus ATCC 53103 under different sodium lactate concentration. (B) The viable count of L. rhamnosus ATCC 53103 under different sodium lactate concentration. [file Image_1.TIF]

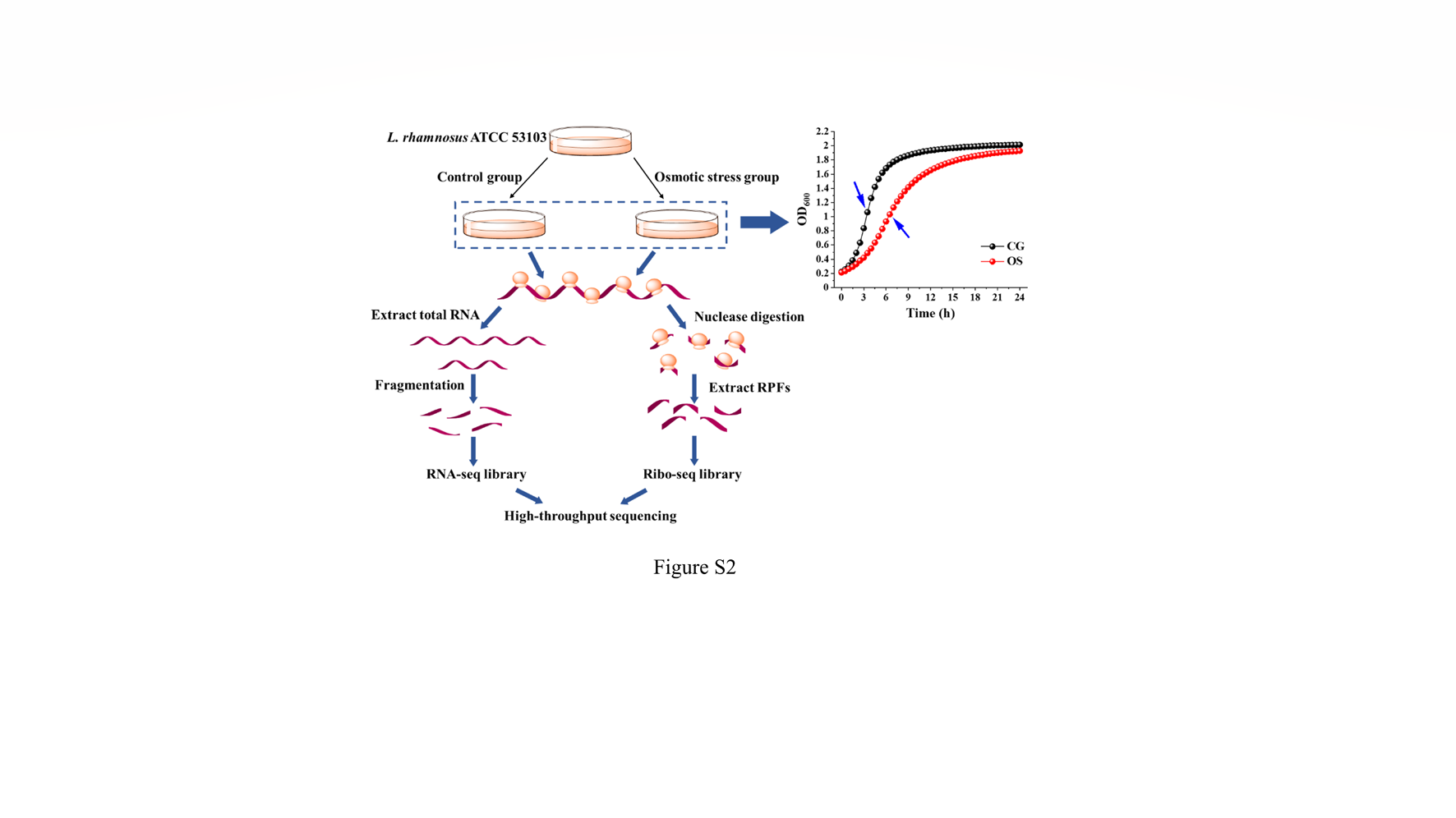

Supplement: Supplementary Figure 2 — Overview of experimental design. RNA-seq and ribosome profiling were performed on CG and OS. [file Image_2.TIF]

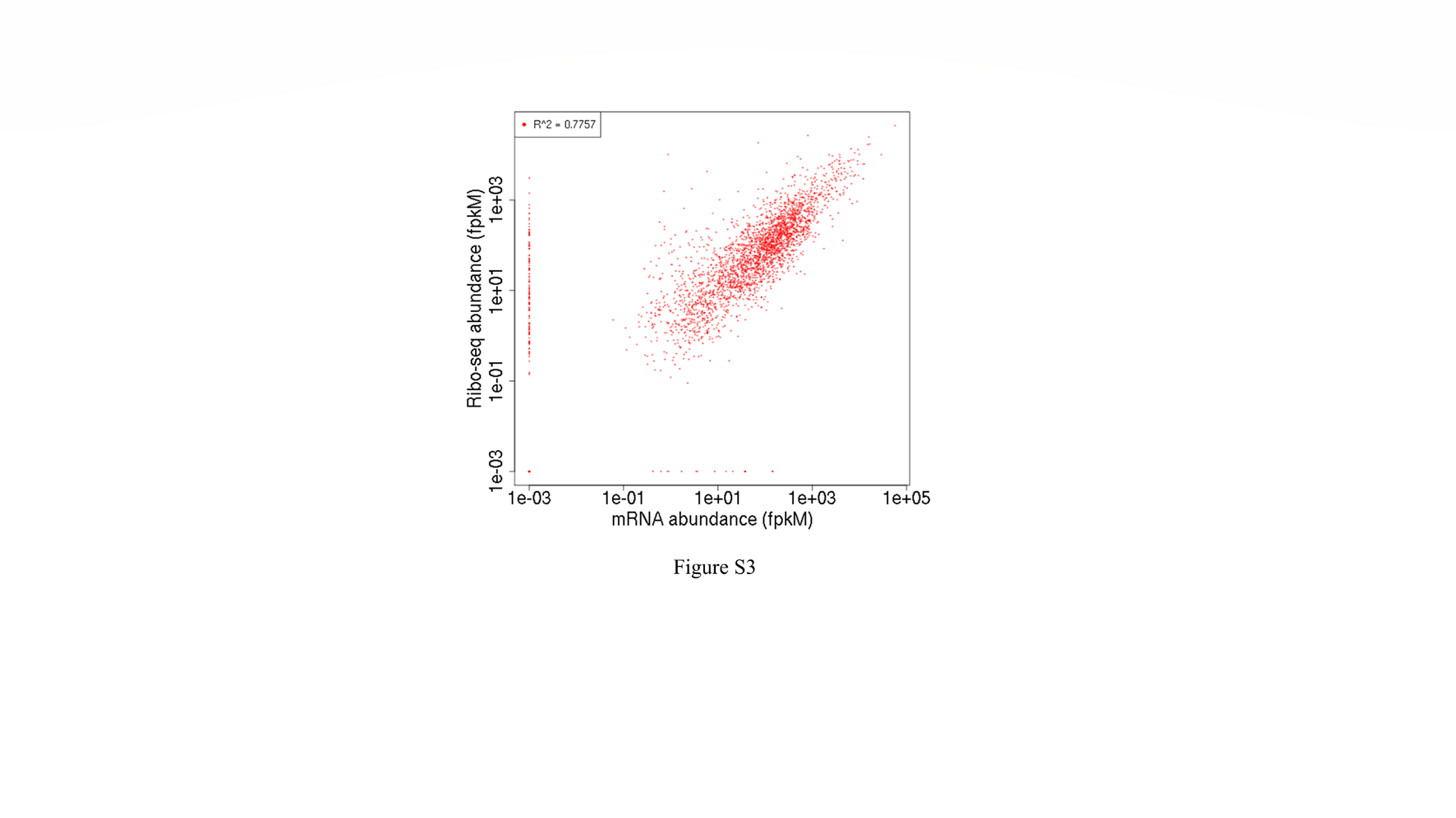

Supplement: Supplementary Figure 3 — Correlation of gene expression of mRNA abundance with Ribo-seq abundance of OS. [file Image_3.TIF]

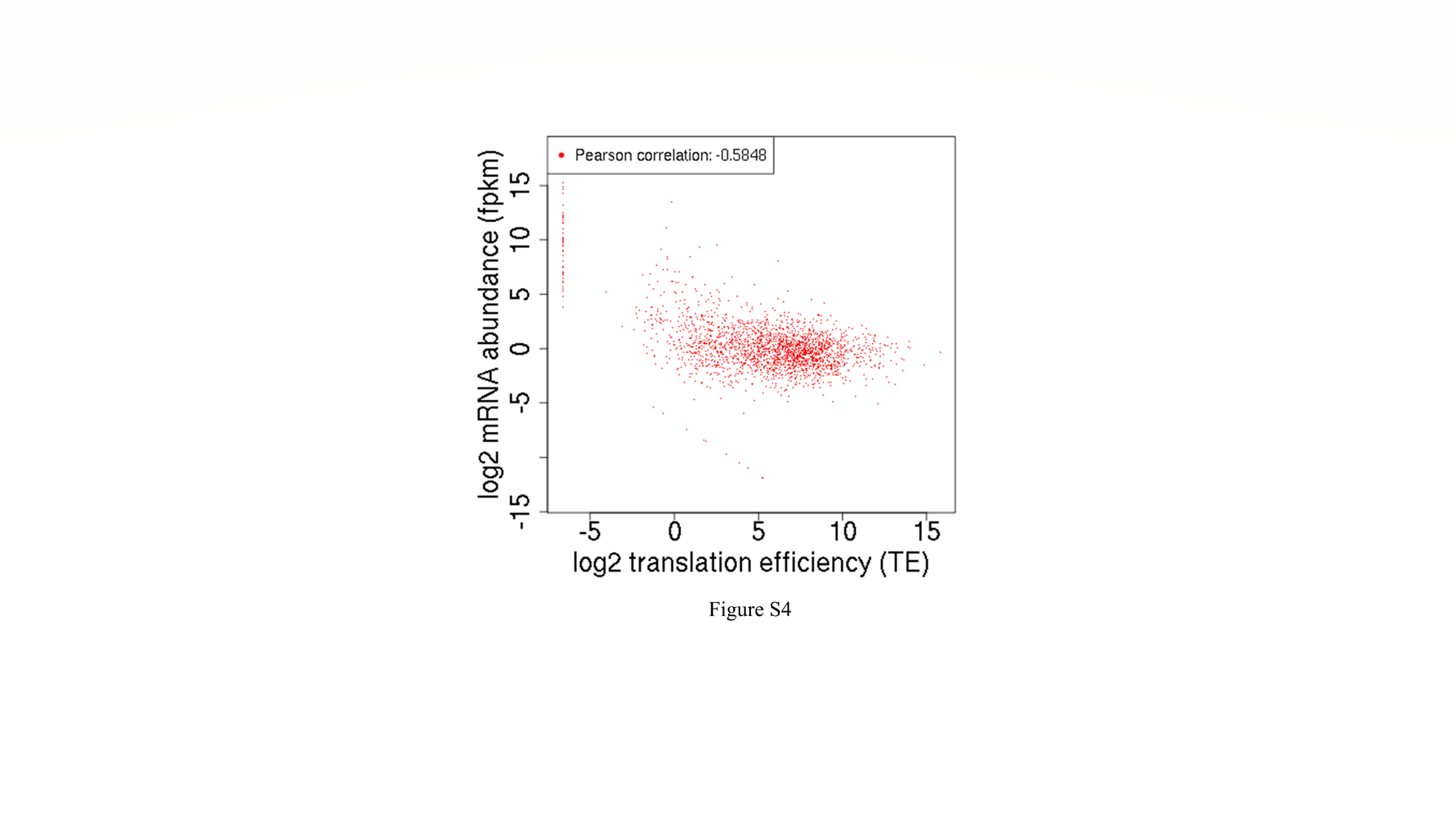

Supplement: Supplementary Figure 4 — Correlation of gene expression of mRNA abundance with gene translation efficiency of OS. [file Image_4.TIF]
