# Supplementary material for: Ribosome Profiling and RNA Sequencing Reveal Genome-Wide Cellular Translation and Transcription Regulation Under Osmotic Stress in Lactobacillus rhamnosus ATCC 53103
Source: Front Microbiol. 2021 Nov 25;12:781454. doi: 10.3389/fmicb.2021.781454 (PMC8656396; doi:10.3389/fmicb.2021.781454)
Supplement: Supplementary file 8 [file Table_4.DOCX]

Table S4 DEGs enrichment result of TE with KEGG pathway analysis.

| Pathway name | Pvalue | Pathway ID | Gene name |
| --- | --- | --- | --- |
| Biosynthesis of secondary metabolites | 0.002 | ko01110 | *trpD, dapB, dapH, lysA, guaB, pmi, tal, cysE, cysK, metB, ldh1, galM, ldh1, ADH2, patB, tpiA, sdaAB, pdhA, pdhB, pdhD, hisE, mvd1, cdsA, rpe, speF, glcK, purD, purH, purN, purK, purE, glgA, glgC, ilvE, accD, accB, dapX, hepT, hepT, hpt, ldh, ldh1, lpdC, argG* |
| Cysteine and methionine metabolism | 0.002 | ko00270 | *cysE, cysK, metB, ldh1, uxS, patB, gshAB, sdaAB, mtnN, ilvE, ldh* |
| Propanoate metabolism | 0.010 | ko00640 | *ldh1, pdhD, pflB, accD, accB, ackA, ldh* |
| Biosynthesis of antibiotics | 0.012 | ko01130 | *trpD, dapB, lysA, pmi, tkt, cysE, cysK, ldh1, galM, ADH2, tpiA, sdaAB, pdhA, pdhB, pdhD, mvd1, acpP, rpe, speF, glcK, purD, purH, purN, purK, purE, spsK, ilvE, rfbC, accD, accB, proB, ldh, argG* |
| One carbon pool by folate | 0.013 | ko00670 | *metF, yqgN, fhs, fmt, purH, purN* |
| Arginine and proline metabolism | 0.019 | ko00330 | *fpaP, speF, nylA, proB, pip* |
| Pyruvate metabolism | 0.034 | ko00620 | *ADH2, pdhA, pdhB, pdhD, pflB, pycB, accD, accB, ackA, ldh, ldh1* |
| Lysine biosynthesis | 0.035 | ko00300 | *dapB, dapH, lysA, pepV, murC, dapX, murF* |
| Metabolic pathways | 0.039 | ko01100 | *cydA, kdgK, uxuA, por, trpD, dapB, dapH, lysA, guaB, ykwC, pmi, manZ, manX, gatB, thiE, tkt, fruA, tal, dhaM, dhaL-1, cysE, cysK, metB, metF, ldh1, lacF, galT, galM, yqgN, luxS, ADH2, pepV, patB, tpiA, ltaS1, atpB, atpH, gshAB, sdaAB, Ldb0724, mraY, murG, mtnN, pdhA, pdhB, pdhD, suhB, fruA, pflB, hisE, pyrE, pyrF, pyrD, pyrC, pyrR1, fhs, mvd1, cdd, cdsA, thiN, rpe, coaBC, rpoZ, speF, glcK, nadD, purD, purH, purN, purK, purE, pycB, coaA, wbgU, glgA, glgC, ilvE, accD, accB, dapX, ackA, ssdA, proB, hpt, ldh, murF, ldh1, lpdC, malL, murQ, nrnA, argG* |
| Biosynthesis of amino acids | 0.066 | ko01230 | *trpD, dapB, dapH, lysA, tkt, tal, cysE, cysK, metB, luxS, pepV, patB, tpiA, sdaAB, mtnN, hisE, rpe, ilvE, THNSL1, dapX, proB, argG* |
| Glycolysis/Gluconeogenesis | 0.096 | ko00010 | *bglA, ldh1, galM, ADH2, tpiA, pdhA, pdhB, pdhD, glcK, ldh, ldh1* |
| Streptomycin biosynthesis | 0.120 | ko00521 | *suhB, glcK, spsK, rfbC* |
| Protein export | 0.120 | ko03060 | *lepB, secG, lspA, yidC* |
| Valine, leucine and isoleucine degradation | 0.140 | ko00280 | *ykwC, pdhD, ilvE* |
| Microbial metabolism in diverse environments | 0.156 | ko01120 | *kdgK, dapB, lysA, tkt, fruA, tal, cysK, metF, ldh1, galM, ADH2, pepV, tpiA, pdhA, pdhB, pdhD, fruA, pflB, fhs, rpe, glcK, rhaD, accD, accB, THNSL1, ackA, ssdA, nylA, ldh, ldh1, nrnA* |
| Carbon metabolism | 0.159 | ko01200 | *kdgK, tkt, tal, cysK, metF, tpiA, sdaAB, pdhA, pdhB, pdhD, fhs, rpe, glcK, accD, accB, ackA* |
| Carbon fixation pathways in prokaryotes | 0.204 | ko00720 | *metF, fhs, accD, accB, ackA* |
| Citrate cycle (TCA cycle) | 0.205 | ko00020 | *pdhA, pdhB, pdhD* |
| Butanoate metabolism | 0.205 | ko00650 | *ADH2, pflB, ssdA* |
| Pantothenate and CoA biosynthesis | 0.205 | ko00770 | *coaBC, coaA, ilvE* |
| Cell cycle - Caulobacter | 0.235 | ko04112 | *clpP, murG, divIB, clpX* |
| Oxidative phosphorylation | 0.252 | ko00190 | *cydA, atpB, atpH, ppaC* |
| Selenocompound metabolism | 0.275 | ko00450 | *metB, patB, trxB* |
| Glutathione metabolism | 0.275 | ko00480 | *GOX1705, gshAB, speF* |
| Ribosome | 0.299 | ko03010 | *rpsD, rpsT, rplU, rpmG, rplL, rplA, rplK, rpmG2, rpmD, rpsQ, rplP, rpsC, rpsS, rpsJ, rpsZ* |
| Tyrosine metabolism | 0.328 | ko00350 | *ADH2, ssdA* |
| Drug metabolism - other enzymes | 0.347 | ko00983 | *guaB, cdd, hpt* |
| Pentose and glucuronate interconversions | 0.353 | ko00040 | *uxuA, por, rpe, rhaD* |
| Peptidoglycan biosynthesis | 0.406 | ko00550 | *murC, mraY, murG, murF* |
| Sulfur metabolism | 0.418 | ko00920 | *cysE, cysK, nrnA* |
| Bacterial secretion system | 0.418 | ko03070 | *secG, yidC* |
| Two-component system | 0.485 | ko02020 | *cydA, ciaR, dltC, desR, yvfT, bceB, citC, ywqE, iphP, htrA* |
| Thiamine metabolism | 0.486 | ko00730 | *thiE, Ldb0724, thiN* |
| Taurine and hypotaurine metabolism | 0.546 | ko00430 | *ackA* |
| Photosynthesis | 0.585 | ko00195 | *atpB, atpH* |
| ABC transporters | 0.632 | ko02010 | *opuCD, opuCC, opuCB, opuCA, expZ, phnC, ftsX, yqgI, potA, potB, potC, bceB, yheI, dppC, bioY, comA, macB2, ecfT, opuCA, macB* |
| Carbon fixation in photosynthetic organisms | 0.663 | ko00710 | *Tkt, tpiA, rpe* |
| Arginine biosynthesis | 0.732 | ko00220 | *argG* |
| Monobactam biosynthesis | 0.732 | ko00261 | *dapB* |
| RNA polymerase | 0.732 | ko03020 | *rpoZ* |
| Glycine, serine and threonine metabolism | 0.754 | ko00260 | *sdaAB, pdhD, THNSL1* |
| Glycerolipid metabolism | 0.754 | ko00561 | *dhaM, dhaL-1, ltaS1* |
| Inositol phosphate metabolism | 0.763 | ko00562 | *tpiA, suhB* |
| Nitrogen metabolism | 0.795 | ko00910 | *cah* |
| Galactose metabolism | 0.814 | ko00052 | *gatB, lacF, galT, galM, glcK, wbgU, malL* |
| Histidine metabolism | 0.841 | ko00340 | *hisE* |
| Purine metabolism | 0.858 | ko00230 | *guaB, guaC, rpoZ, purD, purH, purN, purK, purE, hpt, gppA* |
| Amino sugar and nucleotide sugar metabolism | 0.873 | ko00520 | *Pmi, manZ, manX, galT, glcK, wbgU, glgC, murQ* |
| Pentose phosphate pathway | 0.880 | ko00030 | *kdgK, GOX1705, tkt, tal, rpe* |
| Quorum sensing | 0.902 | ko02024 | *lepB, ciaR, luxS, secG, dppC, comA, yidC* |
| Pyrimidine metabolism | 0.903 | ko00240 | *pyrE, pyrF, pyrD, pyrC, pyrR1, cdd, rpoZ* |
