# Supplementary material for: Ribosome Profiling and RNA Sequencing Reveal Genome-Wide Cellular Translation and Transcription Regulation Under Osmotic Stress in Lactobacillus rhamnosus ATCC 53103
Source: Front Microbiol. 2021 Nov 25;12:781454. doi: 10.3389/fmicb.2021.781454 (PMC8656396; doi:10.3389/fmicb.2021.781454)
Supplement: Supplementary file 9 [file Table_5.DOCX]

Table S5 DEGs regulated on transcription enrichment result of TE with KEGG pathway analysis.

| Pathway | Pvalue | Pathway ID | K_IDs |
| --- | --- | --- | --- |
| Ribosome | 3.81E-04 | ko03010 | *rpsR*, *rpsN*, *rpsO*, *rpmF*, *rpsP*, *rpmA*, *rpmI*, *rplJ*, *rplQ*, *rpsM*, *rplO*, *rpsE*, *rplR*, *rpsH*, *rplE*, *rplX*, *rpmC*, *rplV*, *rplB*, *rpmH* |
| Fatty acid biosynthesis | 0.001 | ko00061 | *accA*, *accC1*, *fabZ*, *fabF*, *bkr2*, *pksA*, *SAS0791*, *fabH* |
| Fatty acid metabolism | 0.001 | ko01212 | *accA*, *accC1*, *fabZ*, *fabF*, *bkr2*, *pksA*, *SAS0791*, *fabH* |
| Phosphotransferase system (PTS) | 0.015 | ko02060 | *SE_1890*, *celA*, *licA*, *gatC*, *fruA*, *manX*, *bglP*, *lacE*, *licA*, *gmuC*, *manX*, *gatC*, *sorB*, *manZ*, *gmuC*, *licA*, *manY*, *manX* |
| Other glycan degradation | 0.017 | ko00511 | *ebgA*, *Fuca1* |
| ABC transporters | 0.059 | ko02010 | *tauB*, *tauA*, *livF*, *ecfA1*, *ecfT*, *tcyJ*, *tcyL*, *HI_1080*, *HI_1079*, *TM_0288*, *potD*, *metQ*, *yxdL*, *znuC*, *gbuC*, *gbuB*, *gbuA*, *cydC*, *mntC*, *ssuB1*, *fetA*, *nodI*, *ugpB* |
| Biotin metabolism | 0.101 | ko00780 | *fabZ*, *fabF*, *bkr2* |
| Prodigiosin biosyntheses | 0.137 | ko00333 | *bkr2*, *pksA* |
| Starch and sucrose metabolism | 0.162 | ko00500 | *SE_1890*, *celA*, *licA*, *treA*, *bbmA*, *licA*, *licA*, *gmuC*, *licA* |
| Toluene degradation | 0.171 | ko00623 | *xylB* |
| Phenylalanine, tyrosine and tryptophan biosynthesis | 0.235 | ko00400 | *trpA*, *trpB*, *aroE* |
| Sulfur metabolism | 0.235 | ko00920 | *tauB*, *tauA*, *ssuB1* |
| Carbapenem biosynthesis | 0.312 | ko00332 | *proA* |
| Biosynthesis of secondary metabolites - unclassified | 0.312 | ko00999 | *acpP* |
| NOD-like receptor signaling pathway | 0.312 | ko04621 | *trxA* |
| Purine metabolism | 0.329 | ko00230 | *purA*, *nrdD*, *xpt*, *nrdE2*, *purM*, *purF*, *purl*, *purQ*, *purS*, *purC*, *add* |
| Pantothenate and CoA biosynthesis | 0.342 | ko00770 | *coaD*, *acpS* |
| Degradation of aromatic compounds | 0.342 | ko01220 | *lpdB*, *xylB* |
| Fructose and mannose metabolism | 0.353 | ko00051 | *sorD*, *fruA*, *manX*, *manX*, *fucI*, *sorB*, *manZ*, *manY*, *manX*, *mtlD* |
| Amino sugar and nucleotide sugar metabolism | 0.383 | ko00520 | *murQ*, *manX*, *nagZ*, *manX*, *sorB*, *manZ*, *manY*, *manX*, *nagB* |
| Taurine and hypotaurine metabolism | 0.430 | ko00430 | *pta* |
| Xylene degradation | 0.430 | ko00622 | *xylB* |
| Plant-pathogen interaction | 0.430 | ko04626 | *glpK* |
| Carbon fixation pathways in prokaryotes | 0.437 | ko00720 | *Pta*, *accA*, *accC1* |
| Pyruvate metabolism | 0.440 | ko00620 | *Pta*, *pdhC*, *oadB*, *accA*, *accC1*, *pox5* |
| Galactose metabolism | 0.480 | ko00052 | *lacC*, *gatC*, *ebgA*, *lacE*, *lacA*, *lacD2*, *gatC* |
| Propanoate metabolism | 0.486 | ko00640 | *Pta*, *accA*, *accC1* |
| Ubiquinone and other terpenoid-quinone biosynthesis | 0.528 | ko00130 | *lpdB* |
| Phenylalanine metabolism | 0.528 | ko00360 | *xylB* |
| D-Alanine metabolism | 0.528 | ko00473 | *ddl* |
| Aminobenzoate degradation | 0.528 | ko00627 | *lpdB* |
| Biosynthesis of unsaturated fatty acids | 0.528 | ko01040 | *bkr2* |
| Two-component system | 0.568 | ko02020 | *dnaA*, *ciaH*, *yxdL*, *glnA*, *citF*, *citD* |
| Ascorbate and aldarate metabolism | 0.585 | ko00053 | *sgbH* |
| Cell cycle - Caulobacter | 0.585 | ko04112 | *dnaA*, *ftsZ* |
| Arginine biosynthesis | 0.609 | ko00220 | *glnA* |
| Monobactam biosynthesis | 0.609 | ko00261 | *dapA* |
| Tyrosine metabolism | 0.609 | ko00350 | *xylB* |
| Base excision repair | 0.635 | ko03410 | *Nth*, *mutM* |
| Nitrogen metabolism | 0.676 | ko00910 | *glnA* |
| Glycerophospholipid metabolism | 0.680 | ko00564 | *glpO*, *plsC* |
| Terpenoid backbone biosynthesis | 0.680 | ko00900 | *mvk* |
| Alanine, aspartate and glutamate metabolism | 0.691 | ko00250 | *purA*, *glnA*, *purF* |
| Citrate cycle (TCA cycle) | 0.732 | ko00020 | *pdhC* |
| Arginine and proline metabolism | 0.778 | ko00330 | *proA* |
| Selenocompound metabolism | 0.778 | ko00450 | *metE* |
| Cysteine and methionine metabolism | 0.782 | ko00270 | *mtaD*, *metE*, *ytsP* |
| Glycine, serine and threonine metabolism | 0.788 | ko00260 | *trpA*, *trpB* |
| Glycerolipid metabolism | 0.788 | ko00561 | *glpK*, *plsC* |
| Glyoxylate and dicarboxylate metabolism | 0.816 | ko00630 | *glnA* |
| Nicotinate and nicotinamide metabolism | 0.816 | ko00760 | *nudC* |
| Homologous recombination | 0.829 | ko03440 | *ssb2*, *ruvA*, *ssb2* |
| Biosynthesis of antibiotics | 0.859 | ko01130 | *trpA*, *trpB*, *dapA*, *aroE*, *pdhC*, *mvk*, *purM*, *purF*, *purl*, *purQ*, *purS*, *purC*, *accA*, *accC1*, *bkr2*, *pksA*, *acpP*, *proA* |
| Biosynthesis of secondary metabolites | 0.929 | ko01110 | *trpA*, *trpB*, *dapA*, *aroE*, *metE*, *glpO*, *xpt*, *pdhC*, *mvk*, *plsC*, *purM*, *purF*, *purl*, *purQ*, *purS*, *purC*, *accA*, *accC1*,*proA*, *lpdB* |
| Quorum sensing | 0.936 | ko02024 | *livF*, *ciaH*, *lacD2* |
| Metabolic pathways | 0.942 | ko01100 | *mtaD*, *trpA*, *trpB*, *dapA*, *murQ*, *purA*, *nrdD*, *ddl*, *iolD*, *lacC*, *gatC*, *thiD*, *fruA*, *manX*, *nagZ*, *ebgA*, *aroE*, *metE*, *nudC*, *glpK*, *lacE*, *lacA*, *bbmA*, *pta*, *gatC*, *xpt*, *pdhC*, *coaD*, *nrdE2*, *mvk*, *plsC*, *glnA*, *purM*, *purF*, *purl*, *purQ*, *purS*, *purC*, *oadB*, *accA*, *accC1*, *fabZ*, *fabF*, *bkr2*, *pksA*, *SAS0791*, *fabH*, *pox5*, *add*, *proA*, *lacD2*, *manX*, *lpdB*, *gatC*, *xylB*, *sgbH*, *sorB*, *manZ*, *manY*, *manX*, *nagB* |
| Peptidoglycan biosynthesis | 0.967 | ko00550 | *ddl* |
| Aminoacyl-tRNA biosynthesis | 0.993 | ko00970 | *gatC* |
| Biosynthesis of amino acids | 0.993 | ko01230 | *trpA*, *trpB*, *dapA*, *aroE*, *metE*, *glnA*, *proA* |
| Carbon metabolism | 0.995 | ko01200 | *Pta*, *pdhC*, *accA*, *accC1* |
| Microbial metabolism in diverse environments | 0.997 | ko01120 | *dapA*, *iolD*, *fruA*, *pta*, *pdhC*, *glnA*, *accA*, *accC1*, *lpdB*, *fucI*, *xylB*, *sgbH* |
| Pyrimidine metabolism | 0.997 | ko00240 | *nrdD*, *nrdE2* |
