# Supplementary material for: Ribosome Profiling and RNA Sequencing Reveal Genome-Wide Cellular Translation and Transcription Regulation Under Osmotic Stress in Lactobacillus rhamnosus ATCC 53103
Source: Front Microbiol. 2021 Nov 25;12:781454. doi: 10.3389/fmicb.2021.781454 (PMC8656396; doi:10.3389/fmicb.2021.781454)
Supplement: Supplementary file 10 [file Table_6.DOCX]

Table S6 DEGs regulated on TE enrichment result of TE with KEGG pathway analysis.

| Pathway | Pvalue | Pathway ID | Gene name |
| --- | --- | --- | --- |
| Cysteine and methionine metabolism | 0.005 | ko00270 | *cysE, cysK, sdaAB, ilvE, ldh, ldh1* |
| Vitamin B6 metabolism | 0.006 | ko00750 | *THNSL1* |
| Glycolysis / Gluconeogenesis | 0.027 | ko00010 | *bglA, galM, tpiA, glcK, ldh, ldh1* |
| Sulfur relay system | 0.030 | ko04122 | *Ldb0724, mnmA* |
| Streptomycin biosynthesis | 0.054 | ko00521 | *suhB, glcK, rfbC* |
| Microbial metabolism in diverse environments | 0.061 | ko01120 | *kdgK, GOX1705, tal, cysK, galM, tpiA*  *fruA, glcK, rhaD, THNSL1, ssdA, ldh, ssdA* |
| Valine, leucine and isoleucine degradation | 0.068 | ko00280 | *ykwC, ssdA* |
| Neomycin, kanamycin and gentamicin biosynthesis | 0.075 | ko00524 | *glcK* |
| Biosynthesis of antibiotics | 0.078 | ko01130 | *GOX1705, tal, cysE, cysK, galM, tpiA, sdaAB, acpP, glcK, ilvE, rfbC, ldh, ldh1* |
| Biosynthesis of secondary metabolites | 0.103 | ko01110 | *GOX1705, tal, cysE, cysK, galM, tpiA, sdaAB, glcK, glgA, ilvE, THNSL1, dapX, hpt, Ldh, ldh1* |
| Carbon metabolism | 0.135 | ko01200 | *kdgK, GOX1705, tal, cysK, tpiA, sdaAB, glcK* |
| Nicotinate and nicotinamide metabolism | 0.142 | ko00760 | *nadD, ssdA* |
| Valine, leucine and isoleucine biosynthesis | 0.145 | ko00290 | *ilvE* |
| Lysine degradation | 0.145 | ko00310 | *ssdA* |
| Biosynthesis of secondary metabolites - unclassified | 0.145 | ko00999 | *acpP* |
| Metabolic pathways | 0.163 | ko01100 | *kdgK, GOX1705, ykwC, manZ, manX, tal, cysE, cysK, galM, yqgN, tpiA, ltaS1, atpB, sdaAB, Ldb0724, suhB, fruA, pyrE, lp_1712, thiN, glcK, nadD, wbgU, glgA, ilvE, THNSL1, dapX, ssdA, hpt, ldh, murF, ldh1* |
| Sulfur metabolism | 0.169 | ko00920 | *cysE, cysK* |
| Bacterial secretion system | 0.169 | ko03070 | *secG, yidC* |
| Inositol phosphate metabolism | 0.197 | ko00562 | *tpiA, suhB* |
| Thiamine metabolism | 0.197 | ko00730 | *Ldb0724, thiN* |
| Protein export | 0.226 | ko03060 | *secG, yidC* |
| Ribosome | 0.237 | ko03010 | *rplU, rplL, rplA, rplK, rpmD, rplP* |
| Biosynthesis of amino acids | 0.266 | ko01230 | *Tal, cysE, cysK, tpiA, sdaAB, ilvE, THNSL1, dapX* |
| Oxidative phosphorylation | 0.312 | ko00190 | *atpB, SH2010* |
| Peptidoglycan biosynthesis | 0.758 | ko00550 | *murF* |
| Alanine, aspartate and glutamate metabolism | 0.794 | ko00250 | *ssdA* |
| Mismatch repair | 0.810 | ko03430 | *mutS2* |
| Homologous recombination | 0.862 | ko03440 | *recR* |
| ABC transporters | 0.888 | ko02010 | *opuCB, potA, potB, opuCA* |
| Quorum sensing | 0.892 | ko02024 | *secG, yidC* |
| Phosphotransferase system (PTS) | 0.947 | ko02060 | *manZ, manX, fruA* |
| Pyrimidine metabolism | 0.968 | ko00240 | *pyrE* |
| Purine metabolism | 0.988 | ko00230 | *hpt* |
