# Supplementary material for: Ribosome Profiling and RNA Sequencing Reveal Genome-Wide Cellular Translation and Transcription Regulation Under Osmotic Stress in Lactobacillus rhamnosus ATCC 53103
Source: Front Microbiol. 2021 Nov 25;12:781454. doi: 10.3389/fmicb.2021.781454 (PMC8656396; doi:10.3389/fmicb.2021.781454)
Supplement: Supplementary file 11 [file Table_7.DOCX]

Table S7 DEGs regulated on homodirectional enrichment result of TE with KEGG pathway analysis.

| Pathway | Pvalue | Pathway ID | *Gene name* |
| --- | --- | --- | --- |
| One carbon pool by folate | 6.95E-05 | ko00670 | *fhs, purH, purN* |
| Carbon fixation pathways in prokaryotes | 2.07E-04 | ko00720 | *Fhs, accD, accB* |
| Biosynthesis of antibiotics | 2.5E-045 | ko01130 | *purD, purH, purN, purK, accD, accB* |
| Biosynthesis of secondary metabolites | 0.001 | ko01110 | *purD, purH, purN, purK, accD, accB* |
| Purine metabolism | 0.001 | ko00230 | *purD, purH, purN, purK* |
| Fatty acid biosynthesis | 0.007 | ko00061 | *accD, accB* |
| Fatty acid metabolism | 0.007 | ko01212 | *accD, accB* |
| Propanoate metabolism | 0.008 | ko00640 | *accD, accB* |
| Carbon metabolism | 0.014 | ko01200 | *fhs, accD, accB* |
| Metabolic pathways | 0.033 | ko01100 | *fhs, purD, purH, purN, purK, accD, accB* |
| Pyruvate metabolism | 0.033 | ko00620 | *accD, accB* |
| Microbial metabolism in diverse environments | 0.126 | ko01120 | *fhs, accD, accB* |
| ABC transporters | 0.642 | ko02010 | *potC* |
