# Supplementary material for: Ribosome Profiling and RNA Sequencing Reveal Genome-Wide Cellular Translation and Transcription Regulation Under Osmotic Stress in Lactobacillus rhamnosus ATCC 53103
Source: Front Microbiol. 2021 Nov 25;12:781454. doi: 10.3389/fmicb.2021.781454 (PMC8656396; doi:10.3389/fmicb.2021.781454)
Supplement: Supplementary file 12 [file Table_8.DOCX]

Table S8 DEGs regulated on opposite enrichment result of TE with KEGG pathway analysis.

| Pathway | Pvalue | Pathway ID | Gene name |
| --- | --- | --- | --- |
| Arginine and proline metabolism | 0.019 | ko00330 | *speF, nylA, proB, pip* |
| Lysine biosynthesis | 0.037 | ko00300 | *dapB, LCABL_00980, dapH, lysA, pepV, murC* |
| Chloroalkane and chloroalkene degradation | 0.058 | ko00625 | *ADH2* |
| Cell cycle – Caulobacter | 0.064 | ko04112 | *clpP, murG, divIB, clpX* |
| Citrate cycle (TCA cycle) | 0.069 | ko00020 | *pdhA, pdhB, pdhD* |
| Pyruvate metabolism | 0.070 | ko00620 | *ldh1, ADH2, pdhA, pdhB, pdhD, pflB, pycB, ackA* |
| Biosynthesis of amino acids | 0.074 | ko01230 | *trpD, dapB, LCABL_00980, dapH, lysA, tkt, metB, luxS, pepV, patB, LCABL_12100, mtnN, hisE, rpe, proB, argG* |
| Biosynthesis of secondary metabolites | 0.080 | ko01110 | *trpD, dapB, LCABL_00980, dapH, lysA, guaB, pmi, tkt, metB, ldh1, ADH2, patB, LCABL_12100, pdhA, pdhB, pdhD, hisE, mvd1, cdsA, rpe, speF, purE, glgC, hepT, hepT, lpdC, argG* |
| Cysteine and methionine metabolism | 0.106 | ko00270 | *metB, ldh1, luxS, patB, gshAB, mtnN* |
| Propanoate metabolism | 0.165 | ko00640 | *ldh1. pdhD, pflB, ackA* |
| Biofilm formation - Escherichia coli | 0.215 | ko02026 | *luxS, glgC* |
| Metabolic pathways | 0.249 | ko01100 | *cydA, uxuA, por, trpD, dapB, LCABL_00980, dapH, lysA, guaB, pmi, gatB, thiE, tkt, fruA, dhaM, dhaL-1, metB, metF, ldh1, lacF, galT, luxS, ADH2, pepV, patB, atpH, gshAB, mraY, murG, mtnN, pdhA, pdhB, pdhD, pflB, hisE, pyrF, pyrD, pyrC, pyrR1, mvd1, cdd, cdsA, rpe, coaBC, rpoZ, speF, purE, pycB, coaA, glgC, ackA, proB, lpdC, mall, murQ, nrnA, argG* |
| Protein export | 0.251 | ko03060 | *lepB, lspA* |
| Peptidoglycan biosynthesis | 0.264 | ko00550 | *murC, mraY, murG* |
| Fatty acid degradation | 0.272 | ko00071 | *ADH2* |
| Carbapenem biosynthesis | 0.272 | ko00332 | *proB* |
| Tryptophan metabolism | 0.272 | ko00380 | *nylA* |
| Styrene degradation | 0.272 | ko00643 | *nylA* |
| Biosynthesis of ansamycins | 0.272 | ko01051 | *tkt* |
| Biofilm formation - Vibrio cholerae | 0.272 | ko05111 | *luxS* |
| Butanoate metabolism | 0.273 | ko00650 | *ADH2, pflB* |
| Pantothenate and CoA biosynthesis | 0.273 | ko00770 | *coaBC, coaA* |
| Pentose and glucuronate interconversions | 0.294 | ko00040 | *uxuA, por, rpe* |
| Terpenoid backbone biosynthesis | 0.294 | ko00900 | *mvd1, hepT, hepT* |
| Selenocompound metabolism | 0.332 | ko00450 | *metB, patB* |
| Glutathione metabolism | 0.332 | ko00480 | *gshAB, speF* |
| ABC transporters | 0.372 | ko02010 | *opuCD, opuCC, opuCA, expZ, phnC, ftsX, yqgI, pstA, bceB, yheI, dppC, oppC, bioY, comA, macB2, ecfT, macB* |
| Taurine and hypotaurine metabolism | 0.379 | ko00430 | *ackA* |
| Plant-pathogen interaction | 0.379 | ko04626 | *tuf* |
| Oxidative phosphorylation | 0.381 | ko00190 | *cydA, atpH, ppaC* |
| Two-component system | 0.394 | ko02020 | *cydA, ciaR, dltC, desR, bceB, citC, iphP* |
| Biosynthesis of antibiotics | 0.395 | ko01130 | *trpD, dapB, lysA, pmi, tkt, ldh1, ADH2, pdhA, pdhB, pdhD, mvd1, rpe, speF, purE, spsK, proB, argG* |
| Ribosome | 0.438 | ko03010 | *rpsD, rpsT, rpmG, rpmG2, rpsQ, rpsC, rpsS, rpsJ, rpsZ* |
| One carbon pool by folate | 0.443 | ko00670 | *metF, fmt* |
| Glycolysis / Gluconeogenesis | 0.515 | ko00010 | *ldh1, ADH2, pdhA, pdhB, pdhD* |
| Arginine biosynthesis | 0.548 | ko00220 | *argG* |
| Tyrosine metabolism | 0.548 | ko00350 | *ADH2* |
| RNA polymerase | 0.548 | ko03020 | *rpoZ* |
| Histidine metabolism | 0.590 | ko00340 | *hisE* |
| Valine, leucine and isoleucine degradation | 0.615 | ko00280 | *pdhD* |
| Nitrogen metabolism | 0.615 | ko00910 | *cah* |
| Pyrimidine metabolism | 0.622 | ko00240 | *pyrF, pyrD, pyrC, pyrR1, cdd, rpoZ* |
| Carbon fixation in photosynthetic organisms | 0.632 | ko00710 | *Tkt, rpe* |
| Carbon fixation pathways in prokaryotes | 0.632 | ko00720 | *metF, ackA* |
| Glycerolipid metabolism | 0.706 | ko00561 | *dhaM, dhaL-1* |
| Microbial metabolism in diverse environments | 0.710 | ko01120 | *dapB, lysA, tkt, fruA, metF, ldh1, ADH2, pepV, pdhA, pdhB, pdhD, pflB, rpe, pflB, nylA, nrnA* |
| Photosynthesis | 0.720 | ko00195 | *atpH* |
| Quorum sensing | 0.731 | ko02024 | *lepB, ciaR, luxS, dppC, comA* |
| Glyoxylate and dicarboxylate metabolism | 0.761 | ko00630 | *pdhD* |
| Nucleotide excision repair | 0.761 | ko03420 | *uvrA* |
| Carbon metabolism | 0.774 | ko01200 | *Tkt, metF, pdhA, pdhB, pdhD, rpe, ackA* |
| Sulfur metabolism | 0.797 | ko00920 | *nrnA* |
| Thiamine metabolism | 0.827 | ko00730 | *thiE* |
| Galactose metabolism | 0.834 | ko00052 | *gatB, lacF, galT, malL* |
| Glycerophospholipid metabolism | 0.875 | ko00564 | *cdsA* |
| Methane metabolism | 0.875 | ko00680 | *ackA* |
| Aminoacyl-tRNA biosynthesis | 0.915 | ko00970 | *glyQ, fmt* |
| Glycine, serine and threonine metabolism | 0.923 | ko00260 | *pdhD* |
| Amino sugar and nucleotide sugar metabolism | 0.926 | ko00520 | *Pmi, galT, glgC, murQ* |
| Purine metabolism | 0.927 | ko00230 | *guaB, guaC, rpoZ, purE, gppA* |
| Pentose phosphate pathway | 0.956 | ko00030 | *Tkt rpe* |
| Alanine, aspartate and glutamate metabolism | 0.959 | ko00250 | *argG* |
| Homologous recombination | 0.982 | ko03440 | *priA* |
| Starch and sucrose metabolism | 0.991 | ko00500 | *glgC, malL* |
| Fructose and mannose metabolism | 0.994 | ko00051 | *Pmi, fruA, mtlA* |
| Phosphotransferase system (PTS) | 0.999 | ko02060 | *gatB, fruA, lacF, mtlA* |
